# Inhibition of *Plasmodium* DNA double strand repair by the combined action of 17-AAG and ML216

**DOI:** 10.3389/fpara.2026.1896831

**Published:** 2026-09-15

**Authors:** Km Tanishka, Wahida Tabassum, Deepak Kumar Rout, Maria Mukherjee, Mrinal Kanti Bhattacharyya

**Affiliations:** Department of Biochemistry, School of Life Sciences, University of Hyderabad, Hyderabad, India

**Keywords:** BLM helicase, DSB repair, homologous recombination, Hsp90, *Plasmodium falciparum*, small molecule inhibitors

## Abstract

The RecQ helicase PfBlm from the human malaria parasite *Plasmodium falciparum* plays a critical role in maintaining genome integrity through its function in homologous recombination mediated DNA repair. Given its essential role in resolving recombination intermediates, PfBlm has emerged as a potential antimalarial drug target. Chemical inhibition of PfBlm helicase activity using the small-molecule inhibitor ML216 sensitize the parasites towards DNA damage inducing agents. Here, we investigated whether inhibition of the molecular chaperone PfHsp90 enhances the antimalarial efficacy of ML216. We demonstrate that the co-treatment with 17-AAG resulted in a marked reduction in the IC_50_ of ML216, indicating potentiation of PfBlm inhibition upon PfHsp90 targeting. We report that PfHsp90 functionally associates with PfBlm and promotes UV irradiation–induced DNA repair by regulating PfBlm stability. Combined inhibition of PfHsp90 and PfBlm caused a pronounced and sustained defect in DNA repair that exceeded the effects of each of the individual inhibitors, consistent with a functional interaction. Together, these findings suggest that PfHsp90 contributes to PfBlm stability and activity, thus provide a rationale for simultaneously targeting PfHsp90 and PfBlm as a strategy to block parasite DNA repair and limit parasite survival.

## Introduction

*Plasmodium falciparum*, the causative agent of the most severe form of human malaria, is continually exposed to genotoxic stress arising from both endogenous metabolic processes and environmental factors throughout its life cycle. During the intraerythrocytic stage, the parasite catabolizes host hemoglobin, releasing free heme, which undergoes oxidation from the ferrous (Fe²^+^) to ferric (Fe³^+^) state (Atamna and Ginsburg, 1993) This oxidative process generates reactive oxygen species (ROS), which are potent inducers of various forms of DNA damage, including double-strand breaks (DSBs). The apparent absence of the classical nonhomologous end joining (C-NHEJ) pathway in *Plasmodium* species highlights the critical role of homologous recombination (HR) as the primary and likely exclusive pathway for DSB repair in these organisms (Roy et al., 2014). This unique reliance on HR underscores the critical importance of DNA repair proteins. Central to the HR process is the Rad51 recombinase, which facilitates the search for homologous DNA sequences and promotes strand exchange- a critical step in accurate DNA repair (Bhattacharyya and Kumar, 2003; Bhattacharyya et al., 2005).

*PfBLM* is the parasite’s homolog of the Bloom syndrome helicase (BLM) from the RecQ family. In this context, RecQ helicases such as *BLM* and *WRN* play vital roles by processing complex DNA structures, stabilizing replication forks, and facilitating accurate DSB repair (Bachrati and Hickson, 2008). Among these two RecQ helicases, *Pfwrn* knockdown causes severe genomic instability with hypermutation, abnormal recombination, and replication defects (Claessens et al., 2018). Whereas *Pfblm* knockout leads to telomere lengthening, relaxed subtelomeric chromatin and upregulation of subtelomeric *var* genes, implying a role in telomere maintenance and suppression of unregulated *var* gene diversification by preserving subtelomeric chromatin organization (Claessens et al., 2018). Functional studies have shown that PfBlm is upregulated in response to DNA damage and interacts directly with key HR proteins such as PfRad51 and PfMre11, suggesting its active involvement in the HR mediated DSB repair (Suthram et al., 2020). Overexpression of wild-type PfBlm enhances survival under genotoxic stress, while the helicase-dead mutant fails to rescue the repair defect, confirming that its helicase activity is essential. Additionally, PfBlm has been implicated in mitochondrial DNA repair, indicating a broader role in maintaining genomic integrity across both nuclear and organellar genomes (Jha et al., 2021). Inhibition of PfBlm with small molecules like ML216 disrupts HR-mediated repair and synergizes with frontline antimalarials, further validating PfBlm as a promising target for therapeutic intervention (Suthram et al., 2020). In humans, Blm participates in recombination-based telomere maintenance in ALT cells (Mendez-Bermudez et al., 2012; Acharya et al., 2014). Using immunoprecipitation and *in vitro* assays the association of telomerase-associated protein 1, Hsp90, and Topoisomerase IIα complex with Blm was identified and such complex was found to co-localize with Blm at telomeric sites that altered the helicase activity of Blm in a substrate-dependent manner (Bhattacharyya et al., 2009). Any such modulation of PfBlm activity by PfHsp90 remained unknown.

Heat shock protein 90 (Hsp90) is a molecular chaperone that governs the folding, stability, and conformational regulation of numerous client proteins. Its activity relies on ATP hydrolysis within its N-terminal Bergerat fold domain (Grenert et al., 1999; Biebl and Buchner, 2019). Pharmacological inhibition of Hsp90 by molecules such as 17-AAG (a geldanamycin analog) or radicicol disrupts this chaperone cycle (Stebbins et al., 1997; Roe et al., 1999). While Hsp90 inhibitors have been extensively explored in cancer therapy for their synergistic effects with DNA-damaging agents, their role in malaria remains less defined. In *P. falciparum*, Hsp90 has been shown to be essential for parasite growth, and chemical inhibition results in marked anti-plasmodial effects (Posfai et al., 2018; Gahlawat et al., 2025). Notably, 17-AAG has been validated to specifically inhibit PfHsp90 activity *in vitro* (Banumathy et al., 2003).

Many Hsp90 client proteins play key roles in stress response pathways (Pennisi et al., 2015). Under genotoxic stress, the proteins responsible for DNA repair and genome maintenance are often Hsp90 clients, relying on the chaperone for stability and proper function (Suhane et al., 2015; Khurana et al., 2016). An earlier study has established that PfSir2, a histone deacetylase essential for subtelomeric heterochromatin formation and *var* gene silencing, is a client of PfHsp90 (Tabassum et al., 2022). Inhibition of PfHsp90 using chemical inhibitors like 17-AAG or Radicicol leads to the depletion of PfSir2A protein levels. Reduced PfSir2A activity leads to loss of silencing at subtelomeric regions, causing de-repression of *var* genes and ribosomal RNA genes and thereby disrupting the parasite’s epigenetic genome regulation (Tabassum et al., 2022).

Similarly, PfRad51 is a bona fide client protein of PfHsp90 (Tabassum et al., 2021b). A dual inhibition of PfHsp90 and PfRad51 produces strong synergistic antimalarial effects by impairing DNA repair. Inhibition of PfHsp90 sensitized parasites to methyl methanesulfonate (MMS)-induced DNA damage, confirming its role in maintaining HR efficiency (Tabassum et al., 2021b). The PfRad51 inhibitor B02 and the PfHsp90 inhibitor 17-AAG showed profound synergism in both drug-sensitive (3D7) and multidrug-resistant (Dd2) parasites (Tabassum et al., 2021b). As evident from this study PfRad51 functions as an Hsp90 client protein, where interaction with Hsp90 is required for efficient DNA repair. Whether a similar regulatory relationship exists between PfBlm and PfHsp90, however, has not been addressed. This raises two central questions: (a) Does PfHsp90 regulate PfBlm abundance and activity during DSB repair in malaria parasites? (b) Can inhibition of PfHsp90 potentiate the antimalarial effect of PfBlm inhibition?

In this study, we demonstrate that the inhibition of PfHsp90 leads to depletion of PfBlm. We further provide evidence of a direct physical interaction between PfHsp90 and PfBlm, confirming that PfBlm is a client of PfHsp90. Notably, simultaneous inhibition of PfHsp90 and PfBlm markedly enhanced parasite susceptibility to DNA damage. These findings suggest simultaneous inhibition of PfHsp90 and PfBlm offers a promising therapeutic approach.

## Materials and methods

### Plasmids

The plasmids pBFM-*PfBLM*, pGADC1: *PfBLM* and pGBDUC1*-PfHSP90* have been previously described (Suthram et al., 2020; Tabassum et al., 2021b).

### Antibodies

Anti-PfBlm was used as described earlier (Suthram et al., 2020). The anti-actin antibody (Sigma, St. Louis, MO) recognizes PfActin, as previously described (Suthram et al., 2020). The anti-Hsp90 antibody (Sigma) recognizes PfHsp90 protein, as previously described (Tabassum et al., 2021a), and the anti-ScActin antibody (Abcam, Cambridge, UK), recognizes ScActin (Suthram et al., 2020; Tabassum et al., 2021b). Horseradish peroxidase (HRP)-conjugated anti-rabbit and anti-mouse secondary antibodies (Promega, Madison, WI) were used.

### Parasite culture

*P. falciparum* 3D7 cultures were grown in RPMI 1640 medium supplemented with 0.5% AlbuMax (Invitrogen, Carlsbad, CA) and 0.005% hypoxanthine, maintained at 5% hematocrit using the candle jar method at 37 °C, as previously described (Trager and Jensen, 2005). Parasite cultures were synchronized at the ring stage by treatment with 5% sorbitol (Sigma).

### Yeast strains

pBFM vector harboring *PfBLM* was transformed into yeast strains iG170Dhsp82 (*MATa can1–100 ade2–1 his3-11*, *15 leu2-3*, *112 trp1–1 ura3–1 hsp82*::*LEU2 hsc82*::*LEU2 HIS3*::*HSP82G170D*) (Nathan and Lindquist, 1995) and W303a (*MATa leu2-3*, *112 his3-11*, *15 ade2-1*, *trp1*, *ura3-1*) (Williams et al., 2005) to generate the strains MMY1 and MMY2, respectively. pGADC1 vector harboring *PfBLM* and pGBUC1 vector harboring *PfHSP90* were transformed into the PJ69-4A strain (*MATa trpl-901 leu2-3*, *112 ura3–52 his3–200 ga14*Δ *ga180*Δ *LYS2*::*GALl-HIS3 GAL2-ADE2 met2*::*GAL7-lacZ*) (Jha et al., 2017) for yeast two-hybrid studies.

### Yeast transformation

Yeast transformation was carried out following previously described protocols (Roy et al., 2014). The pBFM-*PfBLM* plasmid was introduced into wild-type W303a and iG170Dhsp82 strains, and transformants were selected on synthetic complete medium lacking tryptophan (SC-trp). The transformants with the bait plasmids (pGBDUC1 and pGBDUC1*-Pf HSP90*) were selected on SC medium lacking uracil (SC- ura), followed by selecting the transformants with the prey plasmids (pGADC1 and pGADC1-*PfBLM* on SC medium lacking uracil and leucine (SC-ura-leu).

### Yeast two-hybrid analysis

Yeast two-hybrid analysis was performed as previously described (Roy et al., 2014). In brief, 10-fold serial dilutions of PJ69-4A cells co-transformed with bait and prey plasmids were spotted onto synthetic complete medium lacking uracil, leucine, and histidine (SC-ura-leu-his) or lacking uracil, leucine, and adenine (SC-ura-leu-ade) to assess the expression of the *HIS3* and *ADE2* reporter genes, respectively. As a control, cells were also plated on SC medium lacking only uracil and leucine (SC-ura-leu).

### Coimmunoprecipitation

Coimmunoprecipitation was performed using the Pierce cross-link immunoprecipitation kit (Thermo Scientific) following the manufacturer’s protocol as described previously (Tabassum et al., 2021b).

### RNA isolation and real-time PCR

The total RNA was isolated as previously described (Tabassum et al., 2021a) and its concentration was determined by spectroscopic analysis. An aliquot of 5 μg RNA was treated with DNase to eliminate genomic DNA contamination. The absence of residual genomic DNA was verified by PCR using RNA samples that were not subjected to reverse transcription (−RT control). For cDNA preparation, 1 μg of DNase-treated RNA was reverse transcribed using oligo(dT) primers (Sigma, St. Louis, MO, USA) and Omniscript reverse transcriptase (Qiagen, Hilden, Germany). Semi-quantitative real-time PCR was performed using the cDNA. Gene specific primers were used for amplification of *PfBLM* transcripts as described previously (Suthram et al., 2020), and *PfACTIN* was used as the normalizing gene and relative transcript levels were calculated after normalization.

### Western blot analysis

Total protein extracted from the parasite or yeast was separated on an 8% SDS-polyacrylamide gel, and Western blot analysis was conducted as described previously (Roy et al., 2014). The primary antibodies used included anti-PfBlm (raised in rabbits), anti-HsActin, anti-ScActin, and anti-Hsp90 (all raised in mice), applied at a dilution of 1:5,000. HRP-conjugated secondary antibodies (anti-rabbit or anti-mouse) were used at a dilution of 1:10,000. Protein detection was performed using an enhanced chemiluminescence (ECL) kit (Pierce). Band intensity was quantified using Image J software, and the graph was generated using GraphPad Prism 8 software.

### 17AAG and MG132 treatment

Synchronous ring-stage cultures were treated with 1.7 μM 17-AAG for 12 hours, after which 17-AAG was removed, and the parasites were allowed to grow until they reached the late trophozoite stage in complete medium, at which point the culture was harvested. To inhibit proteasomal activity 133 nM MG132 was added to the synchronous ring-stage culture along with 1.7 μM 17-AAG for 12 hours. Following this, the culture was transferred to complete medium, free of 17-AAG and MG132, and allowed to grow until the late trophozoite stage. The parasites were then harvested, and total protein was extracted for western blot analysis.

### PCR-based method to quantify DNA damage

Repair kinetics of damaged nuclear DNA were studied as previously described (Suthram et al., 2020). Briefly, ring stage culture with a parasitemia of 3% was pretreated with different combinations of ML216 (250 nM, 500 nM or 1.0 μM) along with the IC_50_ concentration of 17AAG (510 nM). As described earlier parasites were also treated separately with 510 nM 17AAG or 1.0 μM ML216 as controls (Suthram et al., 2020; Tabassum et al., 2021b). DNA damage was induced at the early trophozoite stage through UV exposure of 100 J/m^2^. Then, the cultures were maintained in the presence of the respective inhibitors for 24 h for recovery. The parasites were harvested for genomic DNA isolation before the damage (UT), just after the damage (0 h), and during the recovery period at 12 h, and at 24 h. PCR amplification of the long-range (7,200 bp) and the short range (269 bp) fragments were performed from equal amounts of isolated genomic DNA for each time point, using the primer sets described earlier (Suthram et al., 2020). Short fragment amplicons were used for the normalization and accordingly the long fragment PCR samples were loaded on the gel and quantified using ImageJ software. The amount of residual damaged before the UV treatment was considered as The amount of residual damaged DNA at each time point was plotted using GraphPad Prism 8 software.

### Determination of IC_50_ of ML216 in presence of IC_50_ of 17AAG

Synchronous 1% ring stage parasite culture was incubated for 48 hours in the presence of IC_50_ concentration of 17AAG (510 nM) along with various concentration of ML216 (500 μM, 200 μM, 100 μM, 50 μM, 20 μM, 10 μM, 1 μM, 0.1 μM, 0.01 μM and 0.001 μM). For each drug combination, triplicate biological repeats were performed. A SYBR green I-based evaluation of parasitemia was done to estimate percent survivability (Tabassum et al., 2021b). Semilogarithmic graphs were plotted using GraphPad Prism 8 software for each combination from three independent experiments to determine the IC_50_ of ML216 in the presence of 17AAG.

## Results

### PfHsp90 physically associates with PfBlm

We sought to investigate if there is any direct physical interaction between PfBlm and the molecular chaperone PfHsp90. To this end we employed a yeast two-hybrid assay, where *PfHSP90* was fused to the *GAL4* DNA-binding domain (bait) harboring a *LEU2* selectable marker and *PfBLM* was fused to the *GAL4* activation domain (prey) harboring a *URA3* selectable marker. The constructs were co-transformed into *S. cerevisiae PJ694a*, and interactions were evaluated based on *HIS3 and ADE2* reporter gene expression. Growth on the selective SC–Leu–Ura–His- plates (top panel), reflecting *HIS3* reporter expression, demonstrated an interaction between PfHsp90 and PfBlm (**Figure 1A**). The lack of growth on SC–Leu–Ura–Ade- plates, however, indicated that the interaction is not strong in nature. The second, the third and the fourth panels acted as negative controls, as they represent the empty-vector controls.

**Figure 1:**
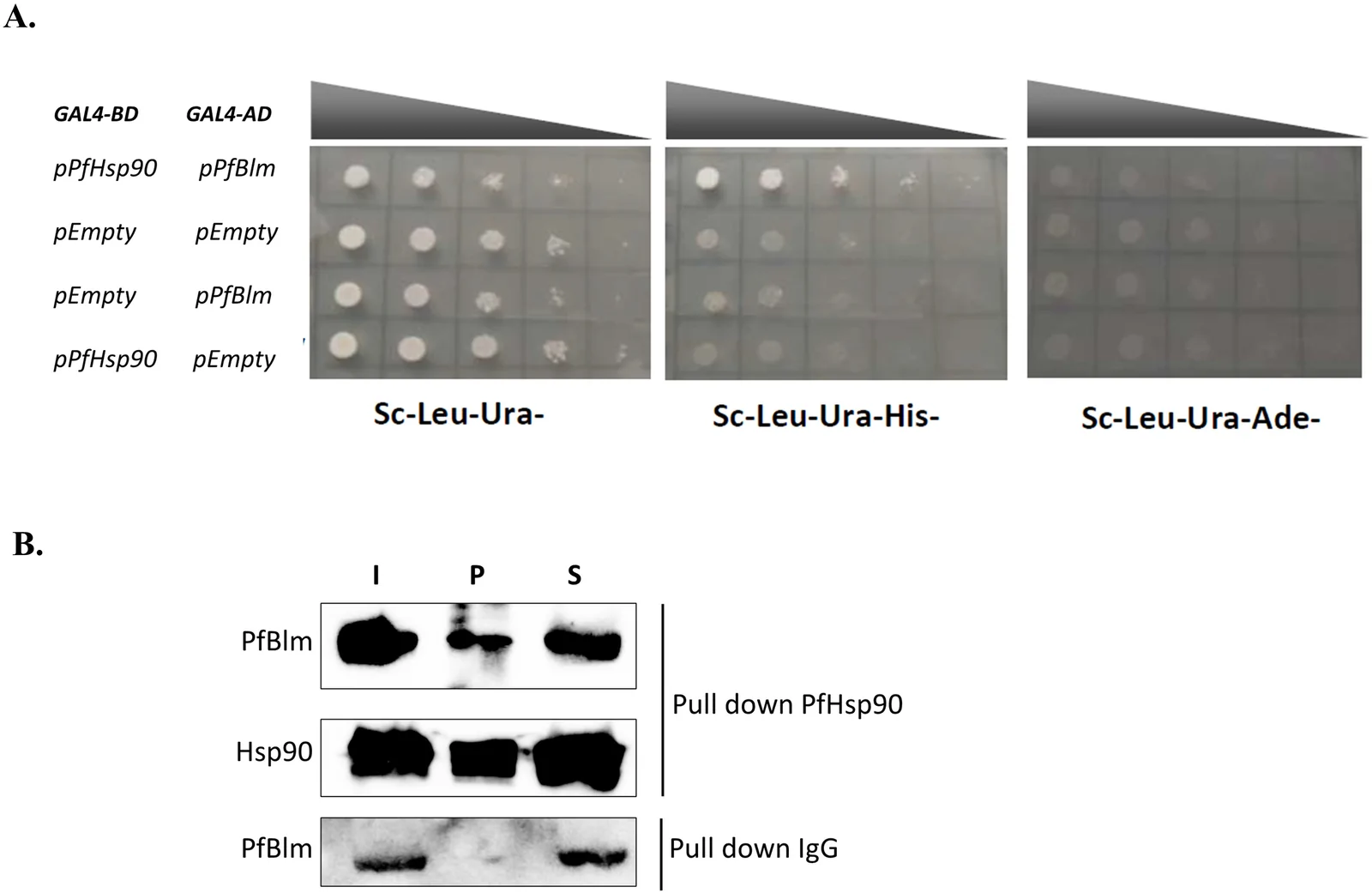
Physical interaction between PfBlm and PfHsp90. **(A)** Yeast two-hybrid assay demonstrating the interaction between PfHsp90 and PfBlm. *PfBLM* was cloned into the pGADC1 vector (GAL4 activation domain), and *PfHSP90* was cloned into the pGBDUC1 vector (GAL4 DNA-binding domain). The yeast strain PJ69-4A, containing *ADE2* and *HIS3* reporter genes, was used for interaction analysis. Cultures were grown to OD_600_ ≈ 0.5, serially diluted 10-fold, and spotted onto dropout media lacking leucine and uracil to confirm plasmid presence. Protein interaction was assessed on plates lacking leucine, uracil, and histidine or adenine. **(B)** Co-IP confirming the interaction between PfHsp90 and PfBlm. Anti-PfHsp90, and IgG antibodies were used to perform the IP. The blots were probed with anti-PfHsp90 or anti-PfBlm antibodies, as indicated. The lanes are I, input; P, IP pellet fraction; and S, supernatant.

To investigate whether such interaction between PfBlm and PfHsp90 also occurs within the malaria parasite, co-immunoprecipitation experiments were performed using *P. falciparum* 3D7 lysates and anti-PfHsp90 antibody. PfBlm was found to be co-immunoprecipitated with PfHsp90, whereas no association was detected when preimmune IgG was used for the immunoprecipitation (**Figure 1B**). In addition to the IP lane (P), PfBlm was also detected in the supernatant (lane S), suggesting that not the entire population of PfBlm were associated with PfHsp90 at the time of immune-precipitation. This result corroborates with the yeast-two-hybrid result and provide further evidence for a physical interaction between the two proteins.

### Inhibition of PfHsp90 leads to the depletion of PfBlm

To check whether the steady-state level of PfBlm is dependent on the chaperone activity of Hsp90, we checked the stability of PfBlm under conditions of Hsp90 functional inactivation. PfBlm was expressed in a temperature-sensitive yeast strain lacking functional Hsp90 (*iG170D hsp82*). In this strain both the endogenous copies of yeast *HSP90*, namely *HSP82* and *HSC82* are deleted and the viability of the yeast strain is maintained by integrating a temperature sensitive allele of hsp82 (*G170D*). Thus, in this strain Hsp90 is functional at the permissive temperature (25°C) and inactive at the non-permissive temperature (37°C).

We found, at the permissive temperature (25 °C), PfBlm remained stable, whereas when shifted to the restrictive temperature (37 °C) for 4 hours, PfBlm levels were markedly reduced, indicating protein destabilization due to loss of Hsp90 function (**Figure 2A**). Band intensity measurement of the western blots from three independent experiments, shows that there is about 75% reduction in the band intensity (**Figure 2B**). No such temperature-dependent effect was observed in wild-type yeast strain W303a when kept at 37 °C for 4 hours (**Figures 2A, B**). In both cases yest actin is used as the loading control. These results confirm that the depletion of PfBlm protein level is due to the loss of Hsp90 chaperone activity, and not due to a temperature effect.

**Figure 2:**
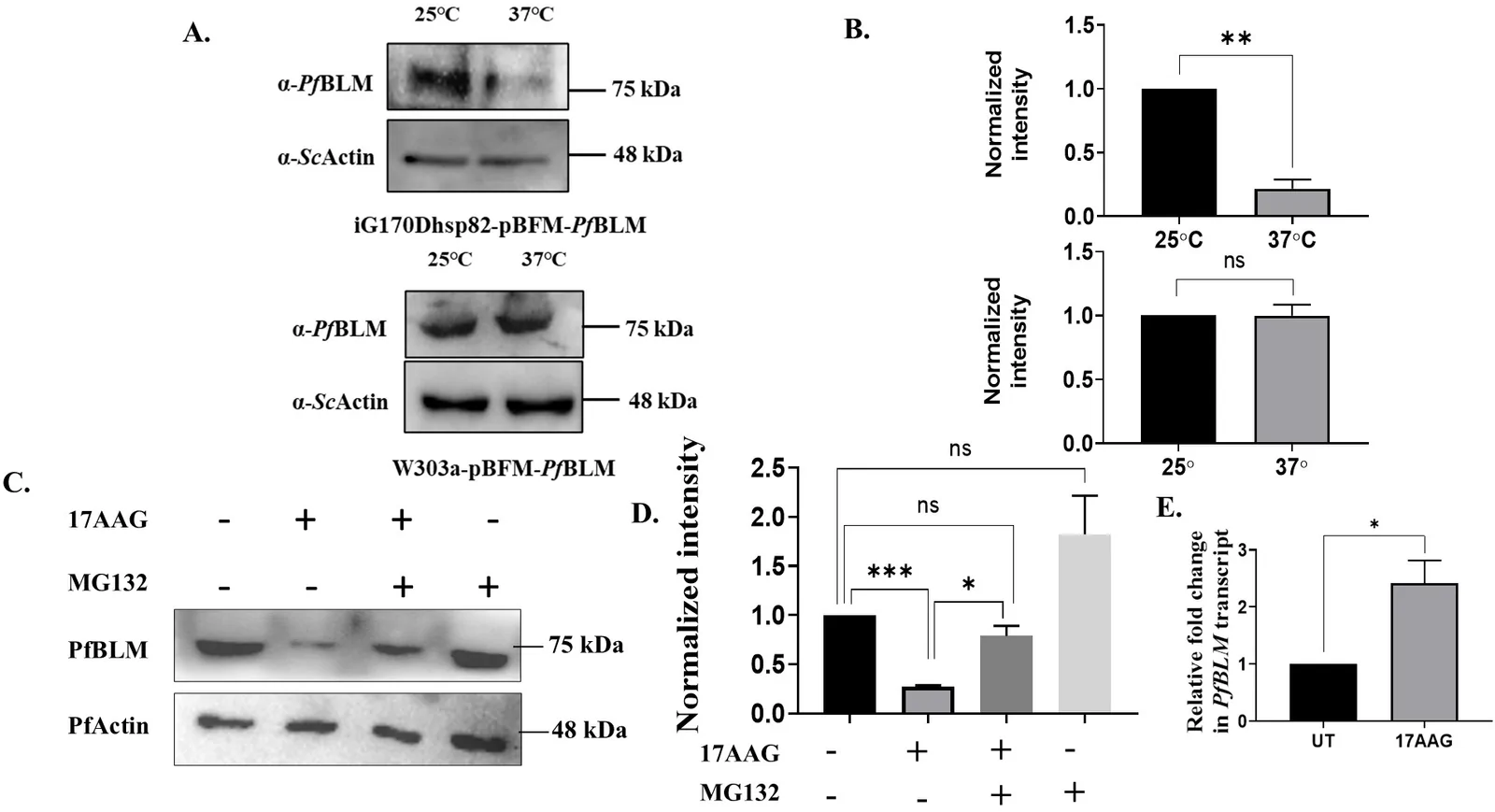
Hsp90 contributes to the stability of PfBlm. **(A)** Western blot analysis of PfBlm expression in total protein extracts from the temperature-sensitive yeast strain iG170Dhsp82 (top), harboring the PfBlm plasmid and grown at either the permissive temperature (25 °C) or restrictive temperature (37 °C). For comparison, protein extracts from W303a yeast strain expressing PfBlm are shown on the bottom. ScActin served as the loading control. Molecular weights of proteins are indicated on the right side of each blot. **(B)** Quantification of PfBlm band intensities from three independent western blots. **(C)** Western blot analysis was performed to assess PfBlm protein levels in *Plasmodium falciparum* parasites under different treatment conditions: untreated (control), treated with 17-AAG (1.7 µM), and co-treated with 17-AAG (1.7 µM), the proteasome inhibitor MG132 (133 nM) and only MG132 (133 nM) treatment. Total protein was isolated from each condition, and actin was used as a loading control to ensure equal protein loading. **(D)** A quantitative graph was generated from band intensities obtained from three independent experiments. **(E)** Graph indicating the semi-quantitative real-time PCR analysis showing mRNA levels of *PfBLM* in control and 17-AAG treated conditions. *PfACTIN* was used as the normalizing control and error bar indicates the SEM obtained from the two independent experiments. Data are presented as mean ± SEM. Statistical significance was determined using a two-tailed Student’s *t* test. Significance levels are indicated as follows: *P = 0.0191 for graph D and *P = 0.0368 for graph E; **P = 0.004; ***P = 0.0002; ns, not significant.

We further investigated the steady state level of PfBlm in *P. falciparum* upon PfHsp90 inhibition. Ring-stage parasites were treated with 1.7 µM of 17-AAG for approximately 12 hours as observed in an earlier study that such treatment was optimum for destabilizing PfHsp90 clients, such as PfRad51 and PfSir2 (Tabassum et al., 2022). Following the treatment parasites were harvested at the late trophozoite schizont stage. Western blot analysis revealed a significant reduction in PfBlm abundance. Importantly, co-treatment with the proteasome inhibitor MG132 restored PfBlm levels despite 17-AAG exposure, while only MG132 treatment resulted in an increase of PfBlm steady-state levels (**Figures 2C, D**). PfActin was used as a loading control, as previously it was found to be not affected by 17-AAG treatment (Tabassum et al., 2021b). This result suggests that the molecular chaperone PfHsp90 is required for the maturation of PfBlm protein and in its absence PfBlm undergoes proteasomal degradation. The increased level of PfBlm when treated with MG132 alone emphasizes that under normal cellular condition the turnover of PfBlm is via the proteasome pathway, and when the proteasome is inhibited the steady-state level of PfBlm is increased.

To determine whether the observed changes in PfBlm were associated with altered transcription, we examined PfBlm transcript levels by semi-quantitative RT-PCR. We found that there is no downregulation in *PfBLM* transcript level upon 17AAG treatment, rather the abundance was significantly increased in the treated parasites compared with the untreated control (**Figure 2E**), indicating transcriptional upregulation of *PfBLM* in response to PfHsp90 inhibition. Thus, the decrease in the steady-state level of PfBlm protein upon PfHsp90 inhibition is due to proteasomal degradation and not due to any transcription down-regulation.

### Inhibitory impact of 17AAG and ML216 cotreatment on DNA repair

PfBlm plays a pivotal role during homology-directed DNA repair in *P. falciparum*. Since PfHsp90 provides stability to PfBlm, it is reasonable to propose that disruption of PfHsp90 activity would compromise PfBlm-dependent DNA repair in this parasite. However, direct correlation between the loss of PfBlm activity due to PfHsp90-inhibition and the loss of DSB repair may not be feasible. As the loss of DSB repair could be due to the depletion of other players of the HR pathway. It has been reported earlier that inhibition of PfHsp90 leads to the depletion of PfRad51 (Tabassum et al., 2021b). Thus, direct inhibition of PfHsp90 alone does not allow selective assessment of PfBlm-dependent repair activity, as the observed DNA repair defects could arise from the loss of PfRad51 function. To specifically evaluate how PfHsp90 inhibition impacts PfBlm-mediated DNA repair process in *P. falciparum*, we employed a drug-combination approach using inhibitors of both PfHsp90 and PfBlm. We therefore examined repair kinetics of UV-induced DSBs in parasites treated with the PfBlm inhibitor ML216 and the PfHsp90 inhibitor 17-AAG. In this experimental framework, an additive effect would indicate that inhibitions of DNA repair by PfHsp90 and PfBlm inhibitors are independent of each other. Whereas a synergistic effect would suggest a functional interdependence, whereby PfHsp90 activity contributes to PfBlm abundance and activity.

To determine the DNA repair kinetics in *P. falciparum*, we used a highly sensitive PCR-based DNA repair assay (Suthram et al., 2020). Synchronous trophozoite-stage parasites were exposed to UV irradiation (100 J/m²) to induce genome-wide DNA double-strand breaks (DSBs). Parasites subjected to UV irradiation restore their genome integrity within 24 h, reflecting efficient DNA repair (**Figure 3A**). Such repair capacity is dependent on functional HR machinery of *P. falciparum* including PfBlm. Earlier it was observed that at 1.7 µM concentration of 17-AAG there was almost 85% depletion in the level of PfRad51 that resulted in 100% inhibition of DSB repair (Tabassum et al., 2021b). However, upon treatment with 420 nM 17-AAG and 850 nM 17-AAG only 18% and 30% depletion of PfRad51 was observed, respectively. Hence, first we sought to determine a particular concentration of 17-AAG that does not completely abrogate DSB repair. We found that treatment with 510 nM 17-AAG does not impair DSB repair and the parasite genome is completely repaired by 24 hours, similar to the no-drug treatment (**Figure 3A**). We then investigated the dose dependent inhibitory effect of ML216 on *Plasmodium* DNA repair. We observed that at 1 µM concentration of ML216 DSB repair was completely inhibited, whereas at 500 nM ML216 concentration there was only 16% inhibition of DSB repair. When parasites were treated with 250 nM ML216, no inhibition was observed (**Figure 3A**). Co-treatment with 510 nM 17-AAG and 500 nM ML216 exhibited a marked increment in the inhibitory effect of the drug-combination. As depicted in **Figure 3B**, there was a 60% inhibition of DSB repair in the presence of 510 nM 17-AAG and 500 nM ML216 drug-combination, which is dramatically higher than both the drugs when used alone. While there was no inhibition when treated with 510 nM 17-AAG, treatment with 500 nM ML216 displayed only 16% inhibition. In case of an additive effect, one would expect that there will be around 16% inhibition only (0% contribution from 17-AAG treatment plus 16% contribution from ML216 treatment). The statistically significant 60% inhibition of the drug-combination implies a strong potentiation. Together, these results demonstrate that combined inhibition of PfBlm and PfHsp90 severely compromises DNA repair in *P. falciparum*, with the combined defect exceeding those observed with either of the inhibitors alone.

**Figure 3:**
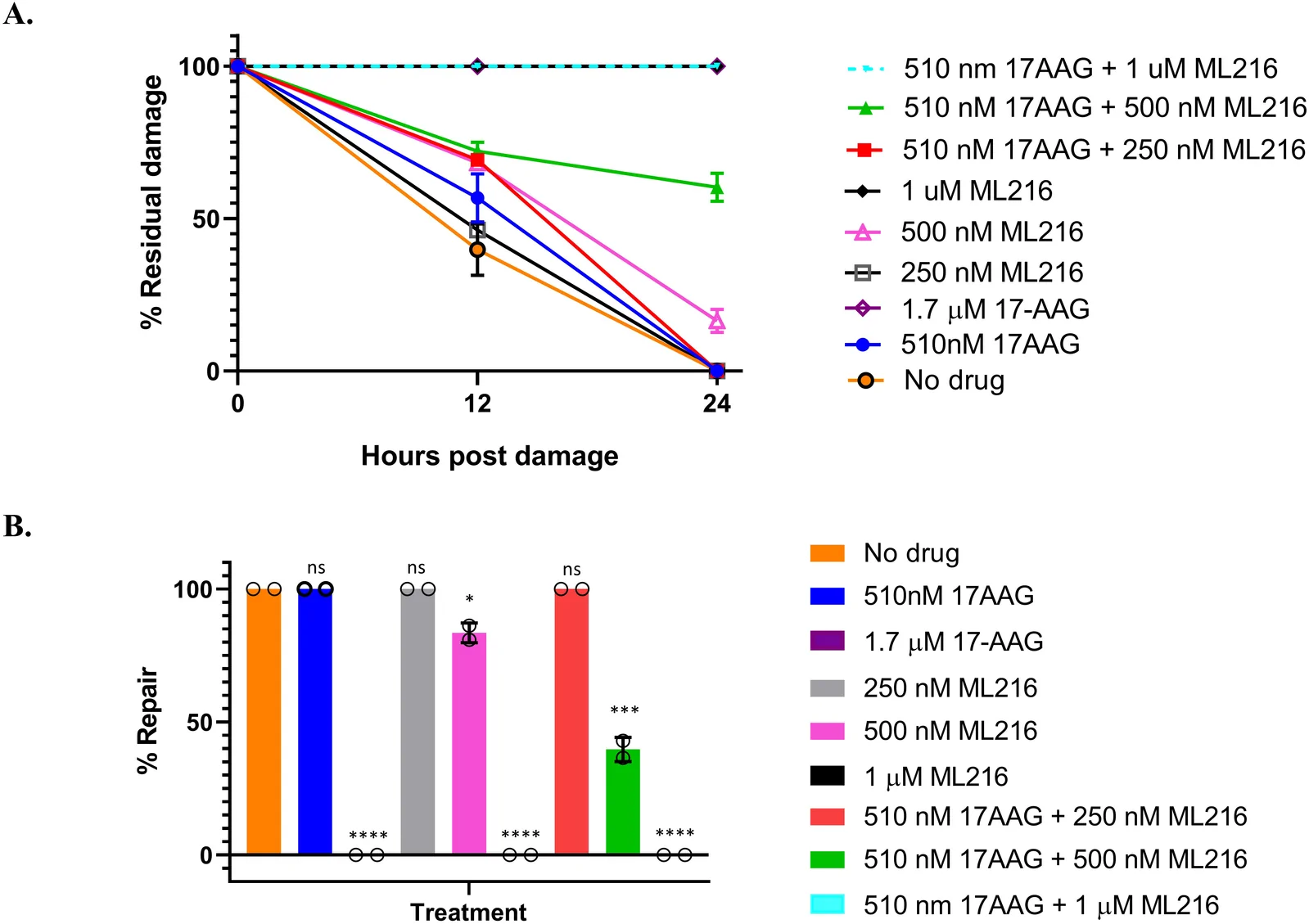
Inhibitory impact of 17AAG and ML216 on PfBlm mediated DNA repair activity. Synchronous *Plasmodium falciparum* 3D7 cultures at the ring stage were pretreated with different combinations of 17-AAG and ML216 for 12 hrs, and then exposed to UV irradiation (100 J/m²). Following UV exposure, cultures were maintained in the respective drugs or drug-free medium for 24 h. Genomic DNA was extracted at the indicated time points: before UV treatment, at 0 h, 12 h, and 24 h post-irradiation followed by PCR-based DNA repair assay. **(A)** The percentage of unrepaired damage and **(B)** Percentage of repair was plotted at the indicated time points using GraphPad Prism software. Data represent the mean ± SEM from three independent experiments. Statistical significance was determined using a one-way ANOVA test. Significance levels are indicated as follows: *P = 0.0115, ***P = 0.0001, ****P < 0.0001.

### Dose dependent potentiation of ML216-inhibitory action by 17AAG

To investigate the potentiation between PfHsp90 and PfBlm inhibitors, we examined the combined effect of 17-AAG and ML216 on parasite growth. Since inhibition of PfHsp90 by 17-AAG destabilizes PfBlm, while ML216 directly targets the helicase activity of PfBlm, we reasoned that partial depletion of PfBlm abundance upon PfHsp90 inhibition would enhance susceptibility of parasites to ML216. Therefore, the IC_50_ values of ML216 were determined in the presence or absence of 17-AAG to assess possible synergistic effects. We treated synchronous trophozoite stage parasite cultures with increasing concentrations of ML216 (0.001 μM to 500 μM) in the presence or absence of 510 nM 17AAG for 48 h, and the IC_50_ values were determined. In the presence of the IC_50_ concentration of 17AAG, the IC_50_ of ML216 decreased from 1.579 μM to 0.041 μM, indicating enhanced parasite sensitivity upon combined treatment. Thus, there was more than 38 fold potentiation of ML216 activity by 17-AAG treatment (**Figure 4**). Since PfHsp90 is required for maintaining PfBlm stability and function, partial inhibition of PfHsp90 is likely to compromise the available pool of functional PfBlm. Consequently, a lower concentration of ML216 is sufficient to inhibit the remaining active pool of PfBlm protein. This finding further supports the notion that PfHsp90 provide clientship to PfBlm and thus, simultaneous inhibition of both PfHsp90 and PfBlm impairs parasite growth.

**Figure 4:**
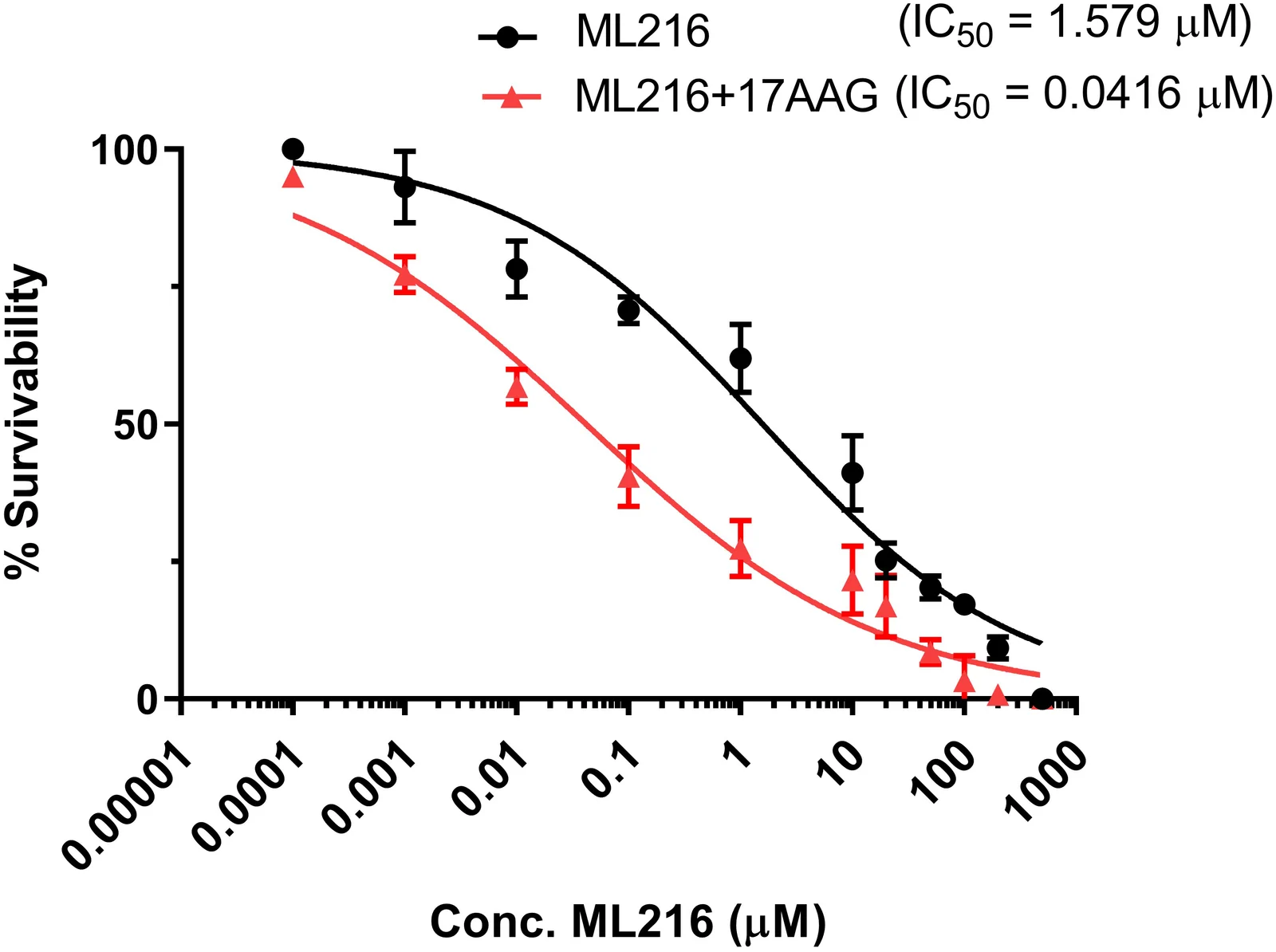
17-AAG potentiates ML216-mediated inhibition of parasite growth. Synchronous Ring-stage parasites (3D7) were grown for 48 h in the presence of various concentrations of ML216 with IC_50_ concentration of 17-AAG (510 nM). Parasite growth inhibition at various concentrations of the drug combination was plotted to obtain the IC_50_ value of ML216 in the presence of IC_50_ concentration of 17AAG. We have plotted a non-linear regression curve: Inhibitor vs. normalized response -Variable slope. For dataset of ML216 alone, the fitted Hill slope was −0.381, with an IC_50_ of 1.579 μM (95% CI: 0.9285 - 2.615) and R² = 0.97. For dataset of ML216 + 17AAG, the fitted Hill slope was −0.330, with an IC_50_ of 0.0416 μM (95% CI: 0.025 - 0.067) and R² = 0.97.

## Discussion

Our findings establish that PfHsp90 confers clientship to PfBlm, highlighting the essential role of PfHsp90 in stabilizing and maintaining the protein homeostasis of RecQ helicase PfBlm in *P. falciparum*. First, we found PfHsp90 physically interacts with PfBlm, indicating chaperone-client relationship. Second, disruption of PfHsp90 activity resulted in proteasome-dependent destabilization of PfBlm, underscoring PfHsp90’s chaperonic role in maintaining proteostasis. Third, the extent of UV induced DNA repair in the presence of Hsp90 inhibitor 17AAG and RecQ helicase inhibitor ML216, is severely compromised. Finally, we observed potentiation of ML216 activity in the presence of 17AAG. Together, these findings demonstrate that PfHsp90 influences the cellular abundance and functional activity of PfBlm.

It was observed in our study that PfHsp90 physically interacts with PfBlm. Similar physical association between human Hsp90 and Blm helicase has been reported in immortalized human cells (Bhattacharyya et al., 2009), indicating a conserved chaperone–helicase interaction that can modulate helicase activity. Human Hsp90 has been shown to regulate the helicase activity of Blm selectively on telomere like DNA substrate (Bhattacharyya et al., 2009). Based on such selectivity of Hsp90, it is speculated that Hsp90 might not act as a constitutive chaperonic foldase for human Blm, rather it may serve as a regulatory factor that stabilizes or activates Blm, only within a particular genomic context. This suggests that, as telomeric regions are prone to replication stress and aberrant recombination such territorial regulation of Blm helicase activity may be critical for maintaining genome stability. Thus, Hsp90-mediated clientship of Blm appears to be dynamically engaged in response to DNA substrate identity, underscoring a broader role for Hsp90 as a molecular hub that coordinates genome maintenance pathways in a context and substrate dependent manner. Whether, such context dependent function of PfHsp90-PfBlm is also operational in *P. falciparum* remains to be uncovered.

In this study it was observed that under PfHsp90 inhibition *PfBLM* was upregulated. This may suggest that PfHsp90 may act as a transcriptional repressor of *PfBLM* expression, probably following a similar mechanism for the regulation of *PfSIR2A* and *PfSIR2B* (Tabassum et al., 2021a).

We observed that Hsp90 inhibitor 17AAG potentiate the action of ML216, which inhibits the activity of PfBlm and leads to reduced viability of parasites. There is a significant inhibition of DSB repair by the combined action of 17AAG and ML216. A plausible interpretation of these results could be that, as PfHsp90 provides clientship to PfBlm, 17AAG treatment destabilizes PfBlm, simultaneously ML216 treatment renders the parasites more susceptible to DNA damage by inactivating the residual amount of PfBlm protein. The co-treatment of 17AAG and ML216 perhaps reduces the cellular levels of active PfBlm protein below a threshold level, insufficient for DSB repair, leading to parasite death. The severe compromise in parasite survival following combined inhibition of PfHsp90 and PfBlm indicated that this drug-combination strategy could confer potent antiparasitic activity.

As reported previously, PfRad51 has been identified as a bona fide client of PfHsp90, with Hsp90 activity required to maintain PfRad51 stability and function during homologous recombination (Tabassum et al., 2021b). PfRad51-mediated recombination has been implicated in the diversification of *var* genes, as recombination events contribute to the generation of novel antigenic variants during asexual blood-stage replication (Vydyam et al., 2023). In this context, PfHsp90 dependent stabilization of PfRad51 is likely to support recombination processes that underlie antigenic variation. In this study, we report PfBlm as a novel client of PfHsp90, extending the repertoire of recombination associated factors under PfHsp90 clientele. In contrast to PfRad51, PfBlm functions primarily as an anti-recombinogenic factor, leading to a negative regulation of *var* gene diversification. Additionally, PfBlm maintaining telomere integrity and preserving subtelomeric chromatin structure where *var* genes are located. It has been shown that loss of PfBlm disrupts subtelomeric heterochromatin, leading to de-repression of *var* loci and altered patterns of antigen expression, suggesting a role for PfBlm in controlling aberrant antigenic variation (Claessens et al., 2018). Together, these finding support a model in which PfHsp90 is likely to function as regulator of antigenic diversification, exerting positive control by stabilizing recombinogenic factor PfRad51, and simultaneously modulating anti-recombinogenic factor PfBlm (**Figure 5**). In addition to keeping a delicate balance between the pro-recombinase and anti-recombinase during *var*-recombination, PfHsp90 also modulates *var*-silencing by providing clientship to PfSir2A in one hand and negatively regulating the transcription of *PfSIR2* on the other hand (Tabassum et al., 2021a; Tabassum et al., 2022). Thus, it appears that PfHsp90 plays important roles during regulation of antigenic variation in *P. falciparum* (**Figure 5**). Such balanced regulation is particularly important in organisms like as *P. falciparum*, where tightly controlled recombination is essential to preserve core genome stability while permitting adaptive processes such as antigenic variation. By balancing the abundance and activities of proteins that promote and restrain recombination, PfHsp90 precisely modulates homologous recombination outcomes rather than promoting DNA-recombination indiscriminately. Such coordinated regulation enables the parasite to maintain genome integrity while allowing controlled antigenic variation, highlighting PfHsp90 as a central regulator of DNA repair driven genome evolution and virulence in *P. falciparum*.

**Figure 5:**
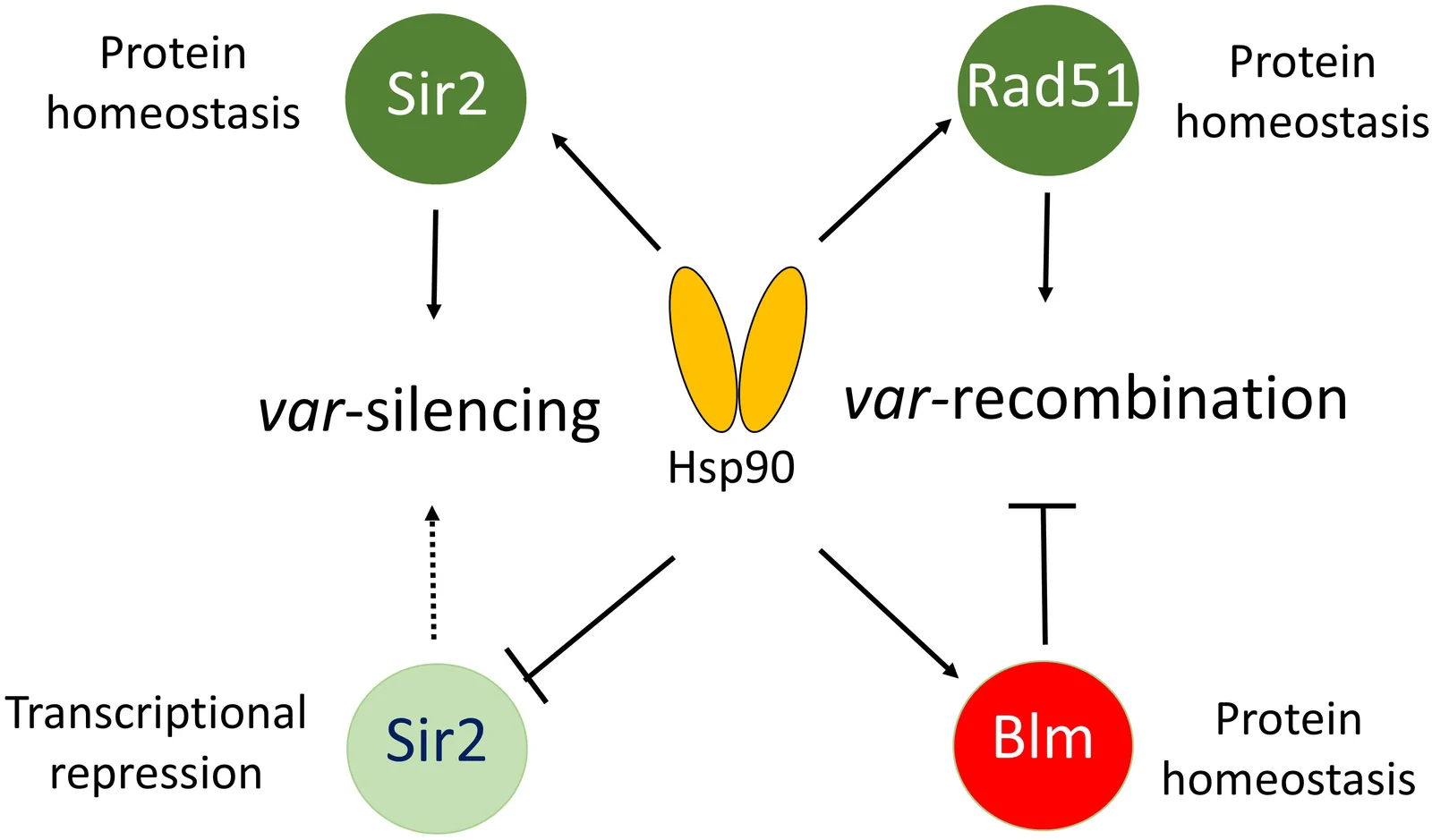
Model depicting a central role of PfHsp90 in regulating antigenic variation. This model highlights the multifaceted roles of PfHsp90 in *var*-gene diversification at the genome level and its transcriptional regulation. PfHsp90 through its chaperone function regulates the stability and functional activity of both the positive and negative regulators of var-recombination, *PfRad51* (Tabassum et al., 2021b) and *PfBlm*, respectively. Through its canonical activity *PfHsp90* facilitates the maturation and stability of *PfSir2A* (Tabassum et al., 2022). Conversely, *PfHsp90* negatively regulates PfSIR2A transcription through its noncanonical nuclear function by occupying the *PfSIR2A* promoter region (Tabassum et al., 2021a) therefore, controlling *var* gene silencing. Thus, PfHsp90 acts as a regulator of both var gene expression and *var* diversification.

In conclusion, given the multifaceted roles of PfBlm in DSB repair and telomere biology and the canonical chaperoning function of PfHsp90, it is reasonable to propose that the PfHsp90-PfBlm axis could be explored for developing a novel chemotherapeutic intervention strategy to curb malaria.

## Data Availability

The original contributions presented in the study are included in the article/supplementary material. Further inquiries can be directed to the corresponding author.
